# Supplementary material for: Ruthenium(II) Complex with 8-Hydroxyquinoline Exhibits Antitumor Activity in Breast Cancer Cell Lines
Source: Cancers (Basel). 2025 Jan 9;17(2):195. doi: 10.3390/cancers17020195 (PMC11763687; doi:10.3390/cancers17020195)
Supplement: Supplementary file 1 [file cancers-17-00195-s001.zip › cancers-3345894-Figure S1.pdf]

Ru(quin)$_2$	T47D			MDA-MB-231		
Control	100	100	100	100	100	100
1 µM	97.5	95.8	98.3	96.5	93.9	97.2
5 µM	94.75	91.2	96.1	92.75	89.5	93.8
10 µM	90	87.3	92.5	87.5	84.1	89
20 µM	85.5	81.2	88.3	80.5	76.4	82
40 µM	75.5	71	78.9	70.25	68.8	75.4
60 µM	65.75	61.3	68.2	62.5	59.1	66.4
80 µM	55.25	51.4	58.1	48	43.3	50.9
100 µM	45.5	44.2	46.8	45.25	43.8	45.9
120 µM	35.25	33.9	36.6	35.4	34	36.2

Ru(quin)$_2$	T47D	MDA-MB-231
Control	100.0	100.0
1 µM	97.2	95.9
5 µM	94.0	92.0
10 µM	89.9	86.9
20 µM	85.0	79.6
40 µM	75.1	71.5
60 µM	65.1	62.7
80 µM	54.9	47.4
100 µM	45.5	45.0
120 µM	35.3	35.2

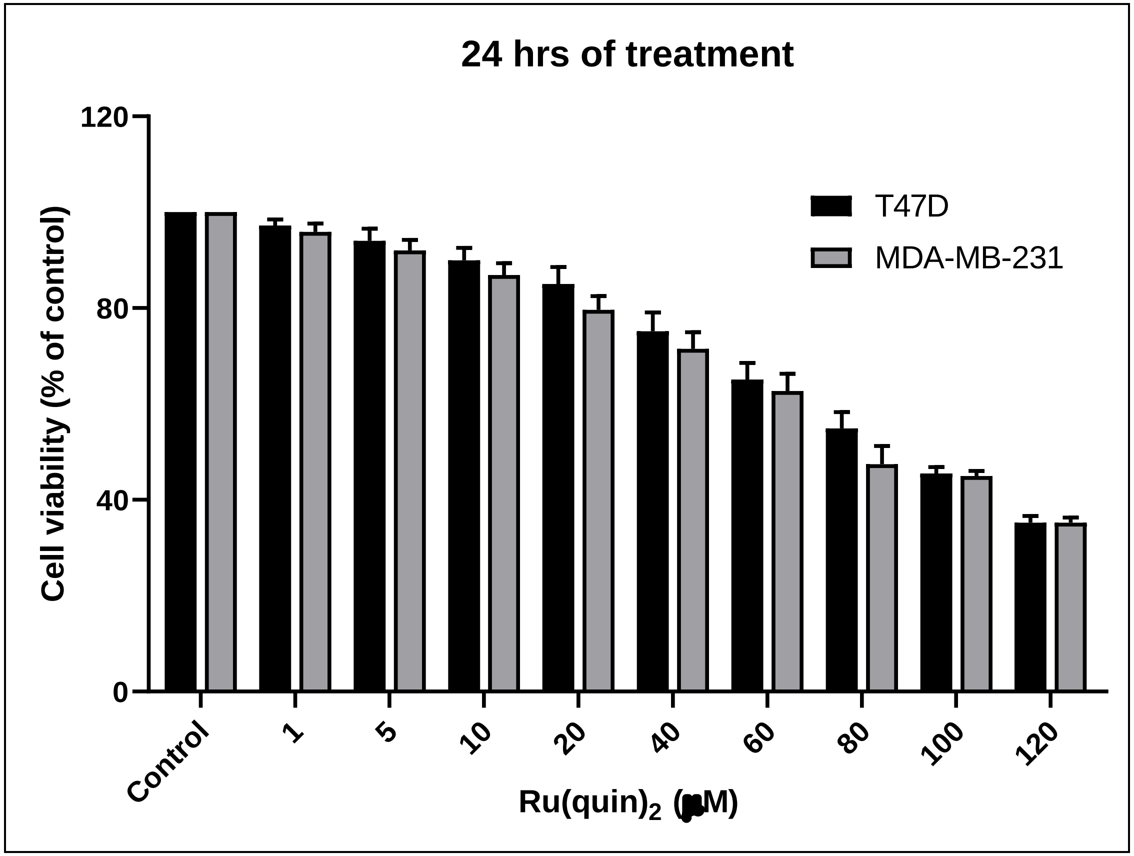

Figure S1. Ru(quin)$_2$ cytotoxic effect in ER+ and TNBC breast cancer cells after 24 h of treatment. Dose-dependent inhibition of cell growth in T47D and MDA-MB-231 cells as assessed by the SRB assay after 24 h of Ru(quin)$_2$ treatment.
