# Supplementary material for: Ruthenium(II) Complex with 8-Hydroxyquinoline Exhibits Antitumor Activity in Breast Cancer Cell Lines
Source: Cancers (Basel). 2025 Jan 9;17(2):195. doi: 10.3390/cancers17020195 (PMC11763687; doi:10.3390/cancers17020195)

Uncropped Western blots from Figure 2

Figure 2D

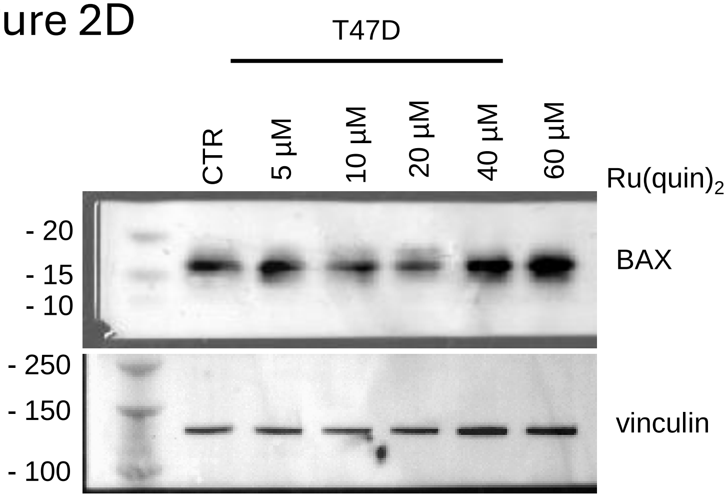

Figure 2E

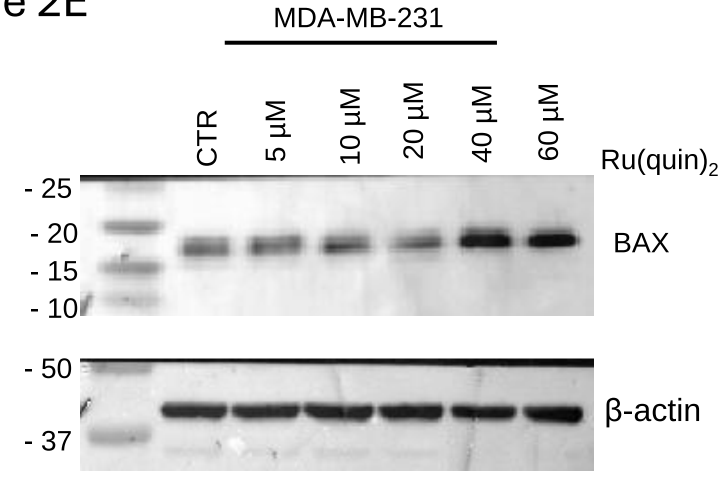

Uncropped Western blots from Figure 3

Figure 3A

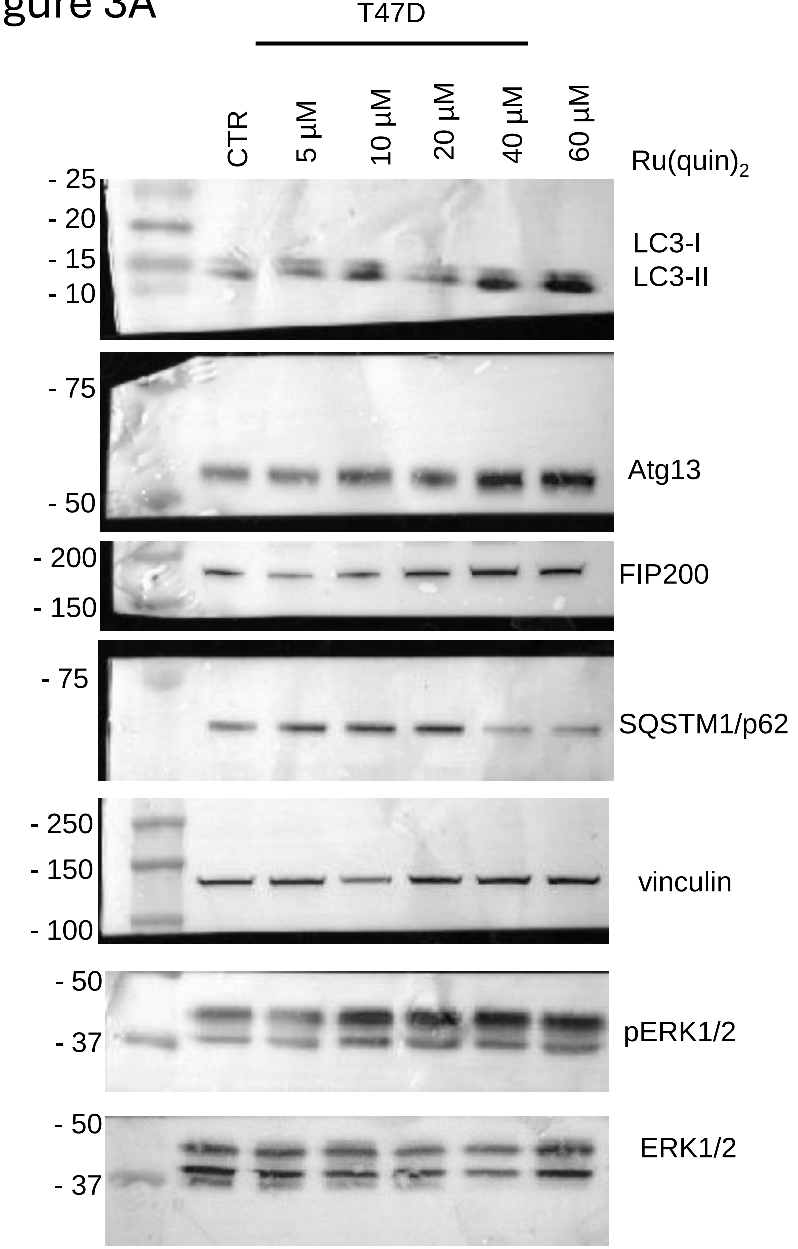

Figure 3B

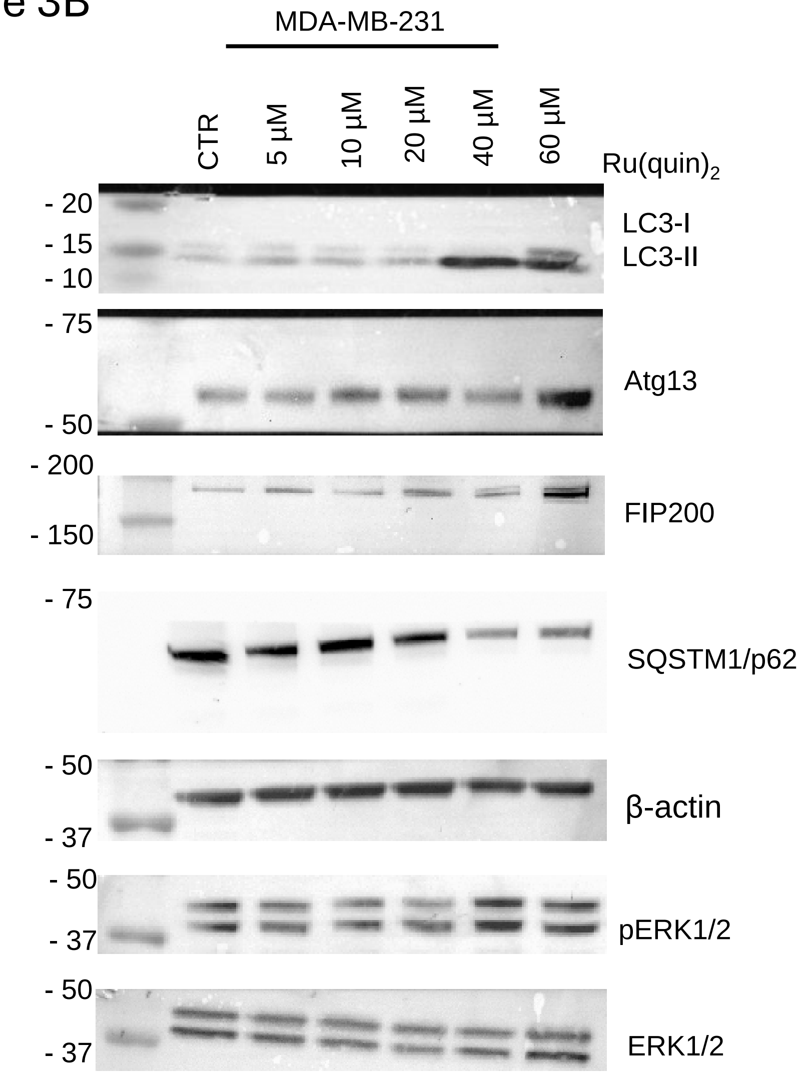

Uncropped Western blots from Figure 4

Figure 4C

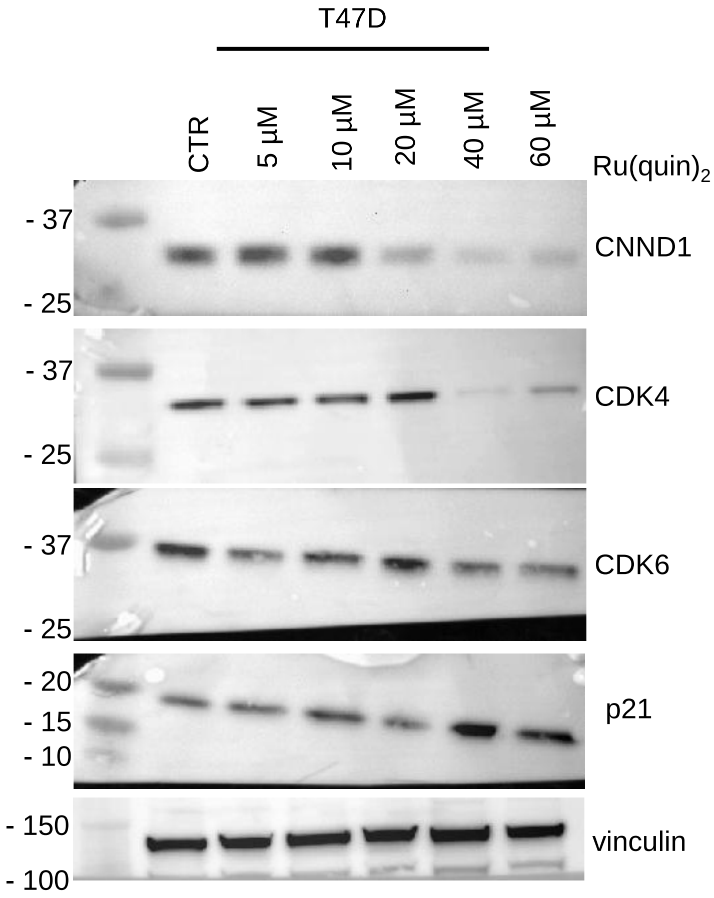

Figure 4D

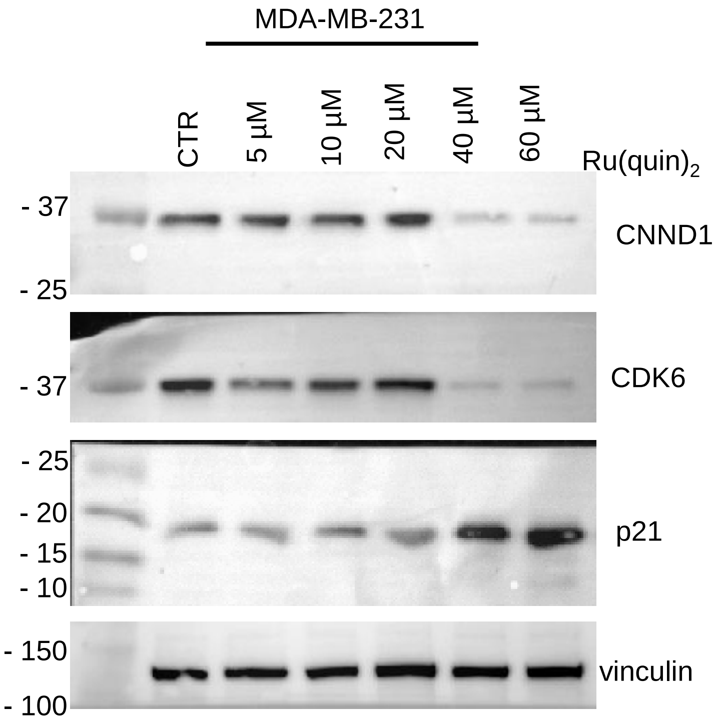

Supplement: Supplementary file 1 [file cancers-17-00195-s001.zip › File S1. original Western blot images.pdf]
